# St. Louis Medico-Chirurgical Society

**Published:** 1880-11

**Authors:** 


### Reports of Societies.

---

### ST. LOUIS MEDICO-CHIRURGICAL SOCIETY.

STATED MEETING JULY 12, 1880.

*Induction of Miscarriage in case of Placenta Prævia.*—Dr. Engleman.—Mr. President, some weeks ago Dr. Boisliniere presented to the Society a placenta which I was requested to examine and report upon. I have several other placentas to show in comparison, which will serve better to explain the one in question. This normrl placenta is one of the same age as that presented by Dr. Boisliniere, and is an excellent contrast to it. I merely relate the history of the case as interesting in itself and somewhat unusual; on June 28th, I was called by Dr. Greiner to see Mrs. B., who deemed herself four months pregnant, and had been flooding almost constantly for two months; Dr. Greiner had given acids, had ordered absolute rest, etc.; in fact, he had exhausted the entire course of treatment which is usually resorted to in such cases, without success; and as the patient was failing, he deemed it necessary, as a dernier resort, to produce miscarriage. The lady had last menstruated four months before I saw her, so that she very naturally concluded that she was four months pregnant Examination revealed the fundus within two inches of the navel; the fetal heart was not to be heard; the placental bruit, however, was distinct; the appearance was that of a pregnancy of from five, to five and one-half months, with which coincided the fact that the patient felt motion, I think, on the 26th, two days before. Again, the internal examination revealed the cervix soft and the os patulous. I suspected placenta prævia, and told her that it would be almost impossible to save the child, that her own life would be endangered if she attempted to go to term; that this could only be accomplished by observing absolute rest in bed and the most perfect quiet. As all other means had been exhausted, I advised, as Dr. Greiner had already done, that a miscarriage be produced. She readily consented, and I introduced the uterine sound, which entered easily, of course, as far as the internal os, where it impinged against a soft yielding mass; this mass was no doubt the placenta; the sound was finally, with some difficulty, passed on one side of the placenta and quite up to the fundus of the uterus. Hot water injections were ordered, and, on the following day, the 29th, a sponge tent was easily introduced a short distance, but impinging against the placenta, it did not pass beyond the internal os. This, of course, had very little effect, and on the following day, the 30th, the external os being enlarged by the first tent, I succeeded in introducing a slippery-elm tent to the fundus, and by its side a sponge tent. Labor pains came on, and twenty-four hours later Dr. Greiner removed the tents; early the next morning the fetus was expelled from the womb; the placenta, however, remained, and this I was called to remove the following morning. The patient made a good recovery. The length of the fetus, which showed signs of life for a few minutes after its delivery, was ten inches or a little over, thus verifying my previous diagnosis that the woman was in her sixth month of pregnancy.

Dr. Thomas has recommended the producing of premature labor in cases of placenta prævia, and speaks of it as resulting very successfully, although he refers more especially to the eighth or ninth month of pregnancy. I think it was in 1877, that he stated he had eleven such cases, in which he had induced premature labor, and had lost only two of the mothers, one from post-partum hemorrhage that set in several hours after delivery, and the other from puerperal fever. Now, I am very sure there was no possibility of preserving the fetus in this case, as the patient had some three and a half or four months to go yet, and she was, moreover, very feeble at this time. The placenta is about three-quarters of an inch in thickness throughout its greatest extent, and where the navel string is inserted, it is over an inch in thickness. Its shape is unusual, being oblong, 4½ inches long, 3½ inches wide at the broadest part, which is at the upper part, and is smooth and regular. The lower part is 2½ inches wide, and very much lacerated. This part lay directly over the internal os, and was undoubtedly lacerated by the action of the sponge tent. The tissue is perfectly healthy and also presents a healthy appearance under the microscope.

*The Placenta in Habitual Miscarriage.*—This specimen which Dr. Boisliniere presented, is from a case of habitual miscarriage, and is itself the result of the fifth premature delivery of the same woman.

He thought after the third accident that an existing inflammatory condition of the uterus might be the cause of miscarriages, and so he treated the patient for this chronic inflammation and succeeded in curing it.

The doctor also thought that there might be another reason for the tendency to miscarriage, namely, syphilitic taint; and although neither the wife nor the husband presented any symptoms of syphilis, he put them both on antisyphilitic treatment. The woman became pregnant for the fourth time, and, under the antisyphilitic treatment, carried the child to near the sixth month, but then an accidant occurred; a carriage in which one of her friends was riding overturned. She felt the shock very much; from that time the doctor found the strength of the child's heart-beats decreasing, and saw that the child was dying by degrees. After her next conception, the fifth, she concealed the fact of pregnancy from him, and was not treated in any way. The result of this last pregnancy is the ovum which is before you.

Attention was called to the interesting fact that these habitual miscarriages, at the same period, are rare, and I will hence relate a similar case, with regard to which I was consulted a few days ago. This patient is a lady twenty-nine years of age, who was married in 1875, and since that time has had five children, one every year; the first one she carried to within two weeks of full term; the next to six and one-half months, delivery taking place in April, 1877; the third to eight months, delivery August, 1878; the fourth to seven and one-half months, delivery July 17th, 1879. Now she is again at about the same period, and, as my informant tells me, she is hourly expecting a miscarriage, because she is now suffering from the same series of symptoms which she has experienced in the previous labors. This patient, in each pregnanay, has been in good health to within two weeks of her confinement, when she is seized with a fever, which continues from ten to thirteen days, and then culminatas in the birth of a macerated fetus, which in every case has presented with the feet.*

Now let us compare the placenta from Dr. Boisliniere's case with the healthy placenta from my case of placenta prævia; they are of nearly the same age, hence we may compare them to advantage. The placenta, the product of Dr. Boisliniere's case of habitual miscarriage, has not the appearance of a healthy one with well developed cotyledons. It has been in alcohol for some two or three months and must be somewhat changed; it is of rather a membranaceous appearance with comparatively undeveloped villi; it is very thin; only three or four cotyledons are still distinct and those are hard, as if infiltrated; the other

*Since making the above remarks, the patient has miscarried, as expected.

The fetus had been born for some weeks. The placenta was much more fully developed than the one presented by Dr. B.; the results of lobular inflammation were very distinct, but not yet so wide-spread; the central portion thin, yellowish and solid, a fibrous mass, not yet contracted, hard, and membranaceous as in Dr. B.'s case.

The appearance of the ovum suggests syphilis, so also the return of the miscarriage, although both parents have so far denied the existence of the poison.

Extremely remarkable is to me the annual recurrence of miscariages in this case, and with almost the same regularity in Dr. B.'s case; again, the unusually large, healthy, well-developed placenta, with inflammation in a single cotyledon, which I have shown, is from a patient in whom I suspect syphilis, and she has also had three children, following each other at intervals of twelve months; she does not miscarry, but bears a healthy child to term and loses it at the age of six or eight months.

portions are dotted with small undeveloped villi. In the centre this placenta is one-third inch in thickness, and gradually decreases towards the margin, so that in two or three places it is not more than one-eighth inch in thickness; towards the centre is an indurated parchment-like surface, with a small, red nucleus towards one extremity. There is no appearance of gummata in the placenta; on the contrary, a more careful examination confirms the statement made by me upon the first hasty examination upon the evening of its presentation, that it was the result of an inflammatory process.*

The placenta bears distinctive marks of placentitis, of

*At the close of this discussion it was ascertained that the husband of the patient whose history is given by Dr. Boisliniere, had been afflicted with venereal diseases at various times before marriage, and had also had a syphilitic ulcer and had been treated for syphilis; at least so he stated to the gentleman who gives the information, whom he consulted, after his engagement, in regard to the propriety of marriage. At that time there was no evidence of syphilis, nor have any signs since appeared, and to Dr. Boisliniere he has denied any infection. I am now convinced of the existence, at some, time of syphilitic infection, on the part of the husband, in both cases of habitual mscarriage, perhaps long before marriage, and to all appearances, completely overcome by treatment. I now also believe that syphilis, latent syphilis of the husband, existed both in Dr. Boisliniere's case and the one related by me, notwithstanding denial by the husbands.

Or could it be latent gonorrhea producing a chronic uterine inflammation and this causing a morbid state of the ovum, especially the placenta? I have distinctly stated, in a previous discussion upon the same subject, that my experience has been a large one, both in hospital and out-door practice in Vienna and Berlin, and has taught me that in case of syphilis of the father, miscarriage would occur, but at a later stage of pregnancy in each following conception, until finally a living child would be born, which would soon die, the next child would live longer, and so on.

I still adhere to these views as they have been forced upon me by oft-repeated observations, but, how to reconcile them to the occurrence of these habitual miscarriages at about the same stage of pregnancy when no direct evidence of syphilis of the father can be obtained, I do not know; unless, indeed, these be the result of latent gonorrhea.

In the cases (of which I have seen so many) cited by me in the previous discussion, distinct evidence of syphilis of the father was at hand, and in those cases the consecutive miscarriages were each at a later stage. At some future time I will elaborate the important and interesting subject upon which I can here give only these crude and hasty thoughts.

simple inflammation with its consequent induration, the pathological condition to which this organ is most frequently subject and most frequently the cause of miscarriage. The different stages of degeneration are distinctly marked in this one specimen, and will show the lobular character of the inflammation by which, one after another, the lobuli disappear until the nutrition of the fetus is completely destroyed.

In the healthy placenta which I have presented, which is of about the same age, we see the full, healthy tufts of the chorion and the cotyledons, though not yet distinctly formed, in the normal condition for that period. In the diseased placenta by its side, there are three of four not entirely shrivelled portions, in which the inflammation has been more recent; they are dense, red and hard, hardened by the fibrinous deposits and reddened by the coloring matter of the blood. Later, this deposit grows paler, and, finally, becomes yellowish red, grayish or whitish; this change in color is consequent upon the change in tissue—which is the same in every one of those inflammatory foci. The young connective tissue, as well as the plastic deposit, gradually contract, and with them is drawn in the destroyed placental tissue; and as a result of the inflammatory change, we have in the last stages, the thin, tough, grayish, callous sheet with no trace of placental tissue left. This is sclerosis of the placenta. As the cotyledons are separately attacked, the nutrition of the fetus is only gradually impaired, and thus the fetus is enabled to survive until the greater part of them are destroyed. We have here [showing a third placenta] a large healthy placenta in which a single cotyledon only has undergone this inflammation; you see upon its upper portion this same yellowish, fibrous, almost membraneous condition. The patient from whom this was taken was delivered of a very large child with a large hard head; it was a very difficult delivery by forceps. Except for the inflammatory degeneration of that one cotyledon, this is an excellent specimen of a healthy placenta at term, and it shows well the lobular nature of placentitis. It is recognized at a glance; the healthy tissue has that peculiar purplish color of the placenta, while the diseased cotyledon presents a yellowish white color. It was evident that there had been a fibrinous deposit, or that an inflammatory process had taken place, and connective tissue had formed.

[Dr. Englemann here presented under microscopic sections of healty placenta injected and showing the villi very perfectly, also sections showing the infiltrated condition of the few cotyledons which are still, in part, preserved in the diseased placenta, and sections from the membranaceous part, showing no villi, but numerous small cells forming new connective tissue. Of these last he said:]

If the process had gone on still longer, it would have led to the formation of perfect connective tissue; as it is, it is already hard, and leather-like. That is the most ordinary pathological condition of the placenta, and there are various ideas with regard to its origin; some think that it is the result of placentitis, which, of course, may itself result from syphilis; others, that it arises from fibrinous deposits in the cotyledons, destroying and compressing the villi, and impairing the nutrition of the fetus. Others still, say that it is due to a hemorrhage in the separate cotyledons, and that we have the same process which we find in hemorrhage occurring in other places; nutrition is impaired, and finally absorption takes place until nothing but connective tissue is left.

Dr. Boisliniere.—The literature on this subject is rather unsatisfactory. The causes are not plain in my mind. One authority says it has an inflammatory origin, a previous placentitis, and that it is followed by the formation of connective tissue. These changes are somewhat analogous to those in the corpus luetum.

Dr. Englemann.—In the corpus luteum we have a fatty degeneration; here, it is fibrinous.

Dr. Boisliniere.—It is something of the stearoid character, a waxy appearance. Perhaps the fatty element is followed by the fibrous element. Rokitansky thinks the yellow color is due to a change of the blood. I have no doubt there was placentitis in this case. Simpson recommends in these cases of recurrent miscarriage a repetition of small bleedings from the arm—take three or four ounces of blood from the arm three or four times a month. He quotes a case which he saved by giving alkaline salts during half the pregnancy, until the formation of the placenta was so complete as to no longer necessitate this treatment. But it must be begun in time and kept up until the placenta is completely formed. The placenta is the fetal organ of respiration, and as a man may live with one lung while the other breathes for two, so long as there are any cotyledons of the placenta left, there are hopes that the child may be carried to a time when it will be possible to induce premature labor and save it. If the child can be carried to seven and a half or eight months, we may accomplish this. It is not unusual thing in uterine pathology to have a recurrence of this trouble in the same woman. The woman's life is always safe in these cases, but we wish to save the life of the child.

Dr. Prewitt—I must confess, I do not see how repeated bleedings, if it be placentitis, would reach the trouble. The question is, where does the fault lie? Does it necessarily come from the mother? Might it not come from the father? The placenta is a fetal organ, and the mother is a rather feeble woman; you would not want to bleed her. I have such a case now, a lady who has miscarried several times. There is some fault with the placenta; there are those hard, white tufts in it, but she is not a subject for depletion. She is rather fleshy, it is true, but rather feeble, and I would not be willing to bleed her; and then, if the fault be in the constitution of the fetus, how would the bleeding of the mother affect the fetus? How would that treatment check the inflammatory action going on in the placenta? Now, if it originates in the condition of the mother, how is the bleeding going to alter that condition?

Very often the fetus itself is diseased. I have noticed that in three or four cases, the fetus was hydrocephalic, and the death of the fetus affected the placenta and destroyed its life, and produced a serious condition of the placenta. The death of the fetus was antecedent to the death of the placenta, so that the disease of the fetus was the cause of the change in the placenta.

There are cases where this process is going on in the placenta before the child is affected. The child dies by inches. In those cases, if you are fortunate enough to see the woman when she is six months gone, the child has been very lively, and the motion is observed to grow feebler and feebler, the child becomes weaker and weaker, anb finally dies in one, two or three weeks. Now did he die from disease of two or three month's duration, or did he gradually, by not calling into use the placenta, cause death of the placenta, cause the placenta to become vitiated and thus destroy nutrition and respiration? That is the question you see. In this case the doctor speaks of, the child was lively enough up to the seventh month, when a runaway accident occurred, and the patient received a shock; from that time the movement of the child diminished, and within two weeks after ceased. The child in that case was hydrocephalic, and how did it come so? Did the shock give the hydrocephalus to the child?

Dr. Engleman.—There was no appearance of hydrocephalus in my case. The child was well preserved. If the child had been dead for a month, it would certainly have been somewhat macerated. It died because the placenta ceased to furnish the necessary nutrition.

Dr. Boisliniere.—It is just the same as a man with consumption. He will live with one lung, in fact, he may live until the last particle of lung dies. This is consumption, phthisis, of the placenta.

The placenta and child die together, and then labor sets in after a greater or less period of time. Simpson, Barnes, and some other writers recommend spoliative bleedings. You ask how the bleeding acts. Congestion precedes the placentitis, and we bleed to take away some of the blood from them.

Dr. Engelman.—Why do you not scarify the uterus?

Dr. Boisliniere.—I would scarify the placenta if I could.

Dr. Prewitt.—There is a difficulty in my mind, to decide whether the placentitis is the result of the faulty inherited constitution of the fetus and its appendage, the placenta, because the placenta is a fetal organ, or originates from the condition of the mother during pregnancy.

If there be extravasation of blood in the placenta, this must come from the fetal circulation, and the bleeding of the mother would not affect that, it strikes me. If the trouble be the faulty constitution of the fetus, it seems to me that bleeding the mother would not reach the difficulty. It might be that the trouble arises from the faulty condition of the mother's system during pregnancy, which would affect the nutrition of the fetus. Then, again, the child might receive the faulty constitution from the father, so that this thing might not occur with another husband.

STATED MEETING, AUGUST 23, 1880.

*Chrysophanic Acid and Pilocarpine in Skin Diseases.*—Dr. Hardaway.—The members present will probably recall to mind a case of psoriasis occurring in a boy of 13, whom I exhibited to the Society some months ago. At that time the eruption had disappeared after a few weeks of treatment by an oitment of chrysophanic acid. He took no constitutional treatment. When seen a few months ago, there had been no return of the eruption. This case is not brought forward to prove that relapses do not occur after the use of chrysophanic acid, for a considerable experience with this drug in this disease demonstrates the contrary; but I mention it merely as exhibiting the now acknowledged remarkable influence of the remedy, and principally to show that local treatment is all sufficient for the relief of psoriasis, and that the recurrence of the eruption is not any more to be expected, than after the most carefully conducted general medication. In a case recently treated, occurring in a gentleman of about 40 years, who had been affected from youth, the eruption was unusually inveterate, and required, for some patches, an unusual strength of ointment —ninety grains to the ounce—for its removal. Usually a ten or twenty per cent. ointment is sufficient. While on this subject, I may mention some recent experiences with the muriate of pilocarpine in skin diseases. I have given the drug in a number of cases of alopecia but as yet with no definite results. In an exceedingly obstinate case of general pruritus, after I had run through the usual list of local and internal remedies without avail, I placed my patient on $\frac{1}{8}$ grain doses of pilocarpine, as advised by Pick. This quantity proving of no service, I increased it to $\frac{1}{4}$ grain twice a day. The former dose had not produced the physiological effects of the drug ; but $\frac{1}{4}$ grain caused profuse perspiration, with but little salivation, and very soon after this effect was noted, the pruritus disappeared.

Dr. Bryson.—I would like to know if there was an increase in the urinary secretion.

Dr. Hardaway.—I made no observations in that regard. The patients seem to differ in regard to the idiosyncracy of the drug. Given in $\frac{1}{8}$ of a grain doses, recently, it produced intense perspiration, while with others $\frac{1}{8}$ of a grain had no effect at all.

Dr, Leete.—When do you give the medicine ?

Dr. Hardaway.—Morning and evening.

*Pilocarpine in Malarial Fever.*—Dr. Pollak.—Last summer, I see, they used pilocarpine in Bellevue Hospital for the epidemic typhoid fever, and in seventeen cases it succeeded remarkably well, one or two injections were sufficient. In no case was there increased secretion of urine. I have used it in adults and small children in malaria.

Dr. Hardaway.—Didn't you find that you had to increase the dose ? That the patient became accustomed to it.

Dr. Pollak.—Very likely they would. In no instance have I given it more than twice, in most of them only once.

Dr. Hardaway.—I omitted to say that after several weeks I found I had to increase the dose.

Dr. Briggs.—How did you administer it ?

Dr. Hardaway.—In water twice a day. In some cases I used as high as a quarter of a grain.

Dr. Gehrung.—Dr. Pollak, in what relation to the malarial attacks did you administer the pilocarpine ?

Dr. Pollak.—I give it just when I have the opportunity. The most fortunate case I have seen, was that of a poor carpenter, who had suffered from malarial fever all summer. When the next rigor commenced, a little chill, I injected at once one-fifth of a grain of pilocarpine, and in less than five minutes it began to influence the attack ; he sat down to dinner with his family and has never had an attack since.

Dr. Gehrung.—Perhaps you gave it accidentally at the right time, that is, shortly before the advent of the chill, in your successful cases, while in the others you may have failed to do so. Or the successful cases may have been those in which the malarial poison has been previously neutralized by the use of quinine, *i. e.* chronic cases, in which not the malaria, but its consequences, as the congestion of certain organs, the habit of recurrence, etc., had to be broken. The unsuccessful cases may have been those in which the malarial poison was not previously neutralized. There is an important question: Is quinine an antidote to the malarial poison, or does it merely promote its elimination? That it is not probably the former is shown by the cures obtained by divers other substances, substances that have apparently no analogy to quinine. Does pilocarpine, besides its other qualities, contain an antidotal principle against malarial? The success following the administration of this substance is probably dependent on its elimenative qualities. Dry heat applied along the spinal column acts very similarly to pilocarpine, and has perhaps some advantages over it. Years ago, when I was yet practicing in Denver, Colorado, where malaria is scarcely ever seen, unless imported, I have treated many of these imported cases by means of dry heat to the spine, with good success; commencing the application about an hour before the expected chill, at times assisted by such small doses of quinine as would otherwise be considered inadequate (two or three one-grain doses), and almost always succeeded in preventing the next attack, though these cases were of every type and degree of severity. Whether the success depended on the peculiar climate of Colorado, or on the treatment used, I have not been able to verify by critical test in this country, as such cases do not come under my observation, since I quit general practice. The heat was applied by means of heated sand bags, eight to ten inches broad and reaching from occiput to coccyx. These bags are placed on a bed made nearly level, and covered with blankets and quilts until the heat transmitted through these feels comfortable to the touch. The patient is made to lie down upon this and covered lightly. Perspiration will soon commence and probably sleep ensue, the chill and the consequent reaction, the fever, does not appear.

Dr. Brigg.—I had long been of the opinion that the Turkish bath was one of the most effectual remedies in malarial disease. I had supposed that the sweating is probably a true eliminative process, by which the system is freed from a certain poison.

Dr. Gehrung.—The poison of malaria, undoubtedly, has the quality of causing contraction of the peripheral capillaries, thus throwing the bulk of the blood into the internal organs, thereby leaving the skin bloodless and causing the chilliness of the surface; then the subsequent reaction dilates the capillaries, and the blood rushes to the surface and perspiration takes place. Now if the heat acts as mentioned before, it will naturally interrupt or prevent the central accummulation of the blood. This being driven at once to the surface, the chill is prevented and reaction not necessary.

Dr. Pollak.—I have frequently applied heat in malaria but not in that form. I used heated bottles, but I never succeeded in preventing the recurrence of the fever.

Dr. Gehrung.—I would especially call attention to the fact that when heat is applied to the spine, care should be taken not to apply heat simultaneously elsewhere. For, if thus applied, it would partly or wholly destroy the beneficial effect, unless it were applied over the whole surface, when it would be peripheral, and not central application of heat.

Dr. Briggs —I am very glad to hear this method spoken of by some one who has successfully experimented with it. It seems to supply us with a means of readily and economically procuring diaphoresis without the administration of medicine or the risk of exposure of the patient.

*Sand Burr in Larynx.*—Dr. Mudd.—I have a little specimen, Mr. President, which may be of interest; it is called a sand burr. A litte child about 10 or 12 years of age playing around the station of Centreville, Ills., rolled from the platform and fell among the bushes, and in falling drew this burr into the larynx. It remained there three or four hours after the accident. Upon looking into the larynx I found the burr suspended in the opening of the larynx. It didn't move with respiration. He was hoarse, but could talk a little and had expectorated some blood. There was some blood about the larynx. I took the curved forceps and made several efforts to catch the burr. I then laid him on his back and made an effort to grasp it, using my finger as a guide, and failed. Dr. Hogden also tried that expedient. We then put him into a chair again. He seemed by this time more tractable and by using the laryngeal mirror and curved forceps which opened antero-posteriorily, I grasped it and lifted it out without difficulty. The boy has had no trouble since. It caused no inflamation of the larynx. It filled pretty well the lumen of the larynx and was held in position by the aryteno-epiglotidean folds.

*Calculus removed by Litholopaxy.*—I have also another specimen here; it is a calculus removed by crushing and and rapid evacuation. It was removed from a young man 26 years of age, who had suffered for six months with symptoms of stone. Sunday, August 8th, we operated upon him. Having anesthetised him and injected five ounces of warm water, we used the ordinary lithontriptor, crushed the stone and evacuated it very readily and quickly by use of Bigelow's evacuator. The patient upon the next day, that is, in the succeeding twenty-four hours, made water twice only, and upon the second day was allowed to go about his business and has been working ever since.

Dr. Pollak.—How much does this weigh?

Dr. Mudd.—About a gramme. The only feature of interest in connection with the operation was the position of the patient, which I think is of some importance and one which I arrived at last winter, in course of some experiments made upon the subject. I believe it is now used by some operator in New York. This position consisted in elevating the pelvis during the crushing, and depressing it during the evacuation. It is certainly a great help in catching the stone. We had no trouble at all in catching it.

Dr. Pollak.—How long did the operation last?

Dr. Mudd.—I should think not more than fifteen minutes.

*Paralysis from injury of the Carotid.*—Dr. Hodgen.—Mr. Chairman, I had recently two cases presenting some very interesting, and to me, unusual conditions. One was a gunshot wound involving the internal carotid as it passes out of the inner end of the petrous portion of the temporal bone opposite the body of the sphenoid. The boy became paralyzed on the opposite side of the body and remained so during the time he lived, two months. In the other case, the right common carotid was wounded about the middle, and this patient had paralysis of the opposite side occurring within the first twenty-four hours, perfect paralysis of motion; and he is still paralyzed, but improving. The accident occurred three weeks ago. In neither case was sensation seriously disturbed. In the last case the patient was able, after the first three days, to move the middle finger also, within a day or two, to flex the ring and little fingers and move the foot and leg of the paralyzed size. The reflex response was very marked in the last case, probably also in the other. I think it is rare for injury of the carotid artery to produce paralysis, yet I see no reason why it should not occur so frequently as to attract attention. There is another interesting feature to which Dr. Mudd called my attention in connection with these cases, that the motor paralysis was complete, sensory paralysis not of so marked a character, if it existed at all, the carotids supplying the anterior and middle portions of the cerebral hemispheres, while the posterior lobes are supplied principally by branches of the vertebrals.—*St. Louis Courier of Medicine.*

